# Oxidative stress changes the effectiveness of artemisinin in *Plasmodium falciparum*

**DOI:** 10.1128/mbio.03169-23

**Published:** 2024-02-07

**Authors:** Camilla Valente Pires, Debora Cassandra, Shulin Xu, Benoit Laleu, Jeremy N. Burrows, John H. Adams

**Affiliations:** 1 Center for Global Health and Interdisciplinary Diseases Research and USF Genomics Program, College of Public Health, University of South Florida, Tampa, Florida, USA; 2 Medicines for Malaria Venture, ICC, Geneva, Switzerland; The George Washington University Milken Institute of Public Health, Washington, DC, USA

**Keywords:** antimalarial agents, malaria, drug resistance evolution, artemisin, oxidative stress

## Abstract

Malaria parasites have adaptive mechanisms to modulate their intracellular redox status to tolerate the enhanced oxidizing effects created by malaria fever, hemoglobinopathies and other stress conditions, including antimalaria drugs. Emerging artemisinin (ART) resistance in *Plasmodium falciparum* is a complex phenotype linked to the parasite’s tolerance of the activated drug’s oxidative damage along with changes in vesicular transport, lipid metabolism, DNA repair, and exported proteins. In an earlier study, we discovered that many of these metabolic processes are induced in *P. falciparum* to respond to the oxidative damage caused by artemisinin, which exhibited a highly significant overlap with the parasite’s adaptive response mechanisms to survive febrile temperatures. In addition, there was a significant overlap with the parasite’s survival responses to oxidative stress. In this study, we investigated these relationships further using an *in vitro* model to evaluate if oxidative stress and heat-shock conditions could alter the parasite’s response to artemisinin. The results revealed that compared to ideal culture conditions, the antimalarial efficacy of artemisinin was significantly reduced in parasites growing in intraerythrocytic oxidative stress but not in heat-shock condition. In contrast, heat shock significantly reduced the efficacy of lumefantrine that is an important ART combination therapy partner drug. We propose that prolonged exposure to intraerythrocytic microenvironmental oxidative stress, as would occur in endemic regions with high prevalence for sickle trait and other hemoglobinopathies, can predispose malaria parasites to develop tolerance to the oxidative damage caused by antimalarial drugs like artemisinin.

**IMPORTANCE:**

Emerging resistance to the frontline antimalarial drug artemisinin represents a significant threat to worldwide malaria control and elimination. The patterns of parasite changes associated with emerging resistance represent a complex array of metabolic processes evident in various genetic mutations and altered transcription profiles. Genetic factors identified in regulating *P. falciparum* sensitivity to artemisinin overlap with the parasite’s responses to malarial fever, sickle trait, and other types of oxidative stresses, suggesting conserved inducible survival responses. In this study we show that intraerythrocytic stress conditions, oxidative stress and heat shock, can significantly decrease the sensitivity of the parasite to artemisinin and lumefantrine, respectively. These results indicate that an intraerythrocytic oxidative stress microenvironment and heat-shock condition can alter antimalarial drug efficacy. Evaluating efficacy of antimalarial drugs under ideal *in vitro* culture conditions may not accurately predict drug efficacy in all malaria patients.

## OBSERVATION

Malaria resurgence during the COVID-19 pandemic increased deaths globally to over 600,000 along with >200 million cases of clinical disease annually. This evident fragility in malaria control occurs in conjunction with continuing emergence of artemisinin resistance (ART-R) in *Plasmodium falciparum* that had already stalled progress in reducing the burden of malaria since 2015 (1). The first genetic marker for ART-R was mutations in *pfkelch13* (2), and more recent evidence shows that this and other types of resistance appear to have spread to highly endemic countries in Africa (3). Artemisinin’s mechanism of action starts with the release of free heme from hemoglobin digestion that acts on the artemisinin endoperoxide bridge to create volatile reactive oxygen species lethal to developing parasites (4, 5). The *pfkelch13* mutation alters the activity of the cytostome, which allows the parasite to tolerate exposure to artemisinin by slowing uptake and digestion of hemoglobin (4, 6). Other resistance mechanisms allow the parasite to tolerate oxidative stress (4, 5, 7) and protein damage (6–9). The incremental spread of K13 resistance mutations and other novel putative ART-R genetic changes suggests there are likely to be fitness costs for the parasite slowing its uptake and/or degradation of hemoglobin (6).

Transcriptional profiling of resistant field isolates reveals similar complex phenotype changes linked to changes in redox metabolism, lipid metabolism, DNA repair, and cellular remodeling (4, 7). Recently, we and others discovered that there is a significant overlap between the parasite’s response to oxidative stress, its survival response to malaria fever, and emerging resistance to the antimalarial drug artemisinin (10, 11). Similarly, the level of malaria protection conferred by the sickle-trait hemoglobin (HbAS) has been associated with increased intraerythrocytic oxidative stress to provide a mechanism of natural protection for HbAS individuals against severe clinical malaria (12). Erythrocytes with sickle cell trait hemoglobin (HbAS), representing individuals heterozygous for healthy adult and sickle hemoglobin, provide strong protection against *P. falciparum* (13, 14).

Based upon these similarities, we hypothesized that the parasite’s adaptive metabolic changes induced by febrile temperatures and intraerythrocytic oxidative stress may alter the sensitivity of *P. falciparum* to antimalarial drugs whose mechanism of action involves oxidative damage to the parasite. We utilized an experimental approach to test for altered drug sensitivity in parasites exposed to intraerythrocytic oxidative stress and heat-shock stress conditions, including use of selected genetic mutants that have altered responses to these stress conditions. The results show that oxidative stress highly significantly increased *P. falciparum* tolerance to artemisinin compounds, while heat shock significantly increased chemosensitivity to lumefantrine.

### Oxidative stress and heat shock shifted the antimalarial chemosensitivity

**Fig 1:**
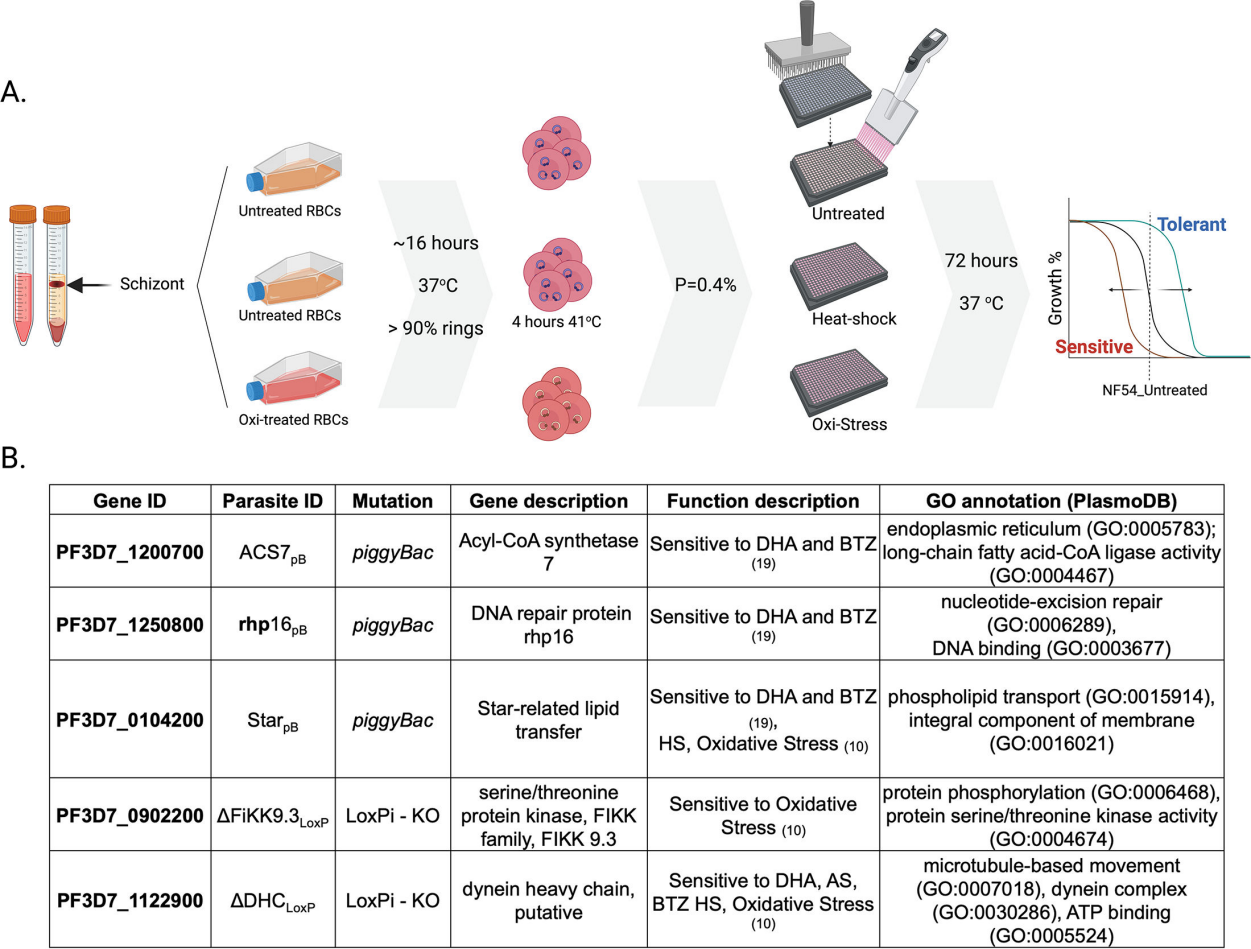
Standardized drug assay to test the effect of oxidative stress and heat-shock condition in the antimalarial chemosensitivity. (A) Schizont stage synchronized parasite lines were split in three groups: one pre-induced oxidative stress (oxi-treated RBCs) and two untreated RBCs, incubated in ideal condition of culture. Approximately 16 hours the parasite reached >90% ring-stages, one of the untreated cultures was incubated to 41°C for 4 hours, mimic fever condition (heat shock [HS]); then, the parasitemia was adjusted to 0.4%, and the parasites were spread on plates with due dilution of drug (Table S1) and incubated for 72 hours in normal conduction of cultures. Plates were analyzed by first reading on the CLARIOstar plate reader for relative fluorescence units at the optimal SybrGreen emissions. The relative IC_50_ values were calculated by interpolation of the probit transformation of the log(dose)-response curve. Each batch of mutant assays was accompanied with wild-type NF54. All compounds were performed in three to five biological replicates (Table S2). (**B)** Mutant’s line information: disrupted gene IDs, parasite IDs, type of mutations, gene and function descriptions, and Gene Ontology (GO) annotation (PlasmoDB).

The effects of oxidative stress and heat shock on the parasite’s sensitivity were evaluated with the artemisinin compounds qinghaosu (QHS) and dihydroartemisinin (DHA) along with other common antimalarial drugs and lead compounds (Table S1). In addition to wild-type NF54, we included selected isogenic genetic mutants (*piggyBac* and targeted mutations) with alterations in lipid metabolism, cellular trafficking and remodeling, and DNA metabolism pathways (4, 5, 7, 8, 15), which are vital to the parasite’s survival responses to artemisinin and heat shock (10, 16, 17) (Fig. 1). The parasites were grown in parallel in red blood cells (RBCs) chemically treated [supplemental material, “Red blood cells (RBC) Oxidative Stress Pre-treatment”] to create an intraerythrocytic oxidative stress microenvironment (Oxi_group) like HbAS (12), and untreated RBCs grown under ideal culture conditions as a control (supplemental material). A third group of parasites was incubated at ring stages for 4 hours to 41°C (heat shock, HS_group) (Fig. 1A), which mimics the malarial fever occurring *in vivo*. The parasites were tested for altered sensitivity to QHS, DHA, lumefantrine, mefloquine, the proteasome inhibitor bortezomib (BTZ), and Medicines for Malaria Venture (MMV) lead compounds (Table S1).

**Fig 2:**
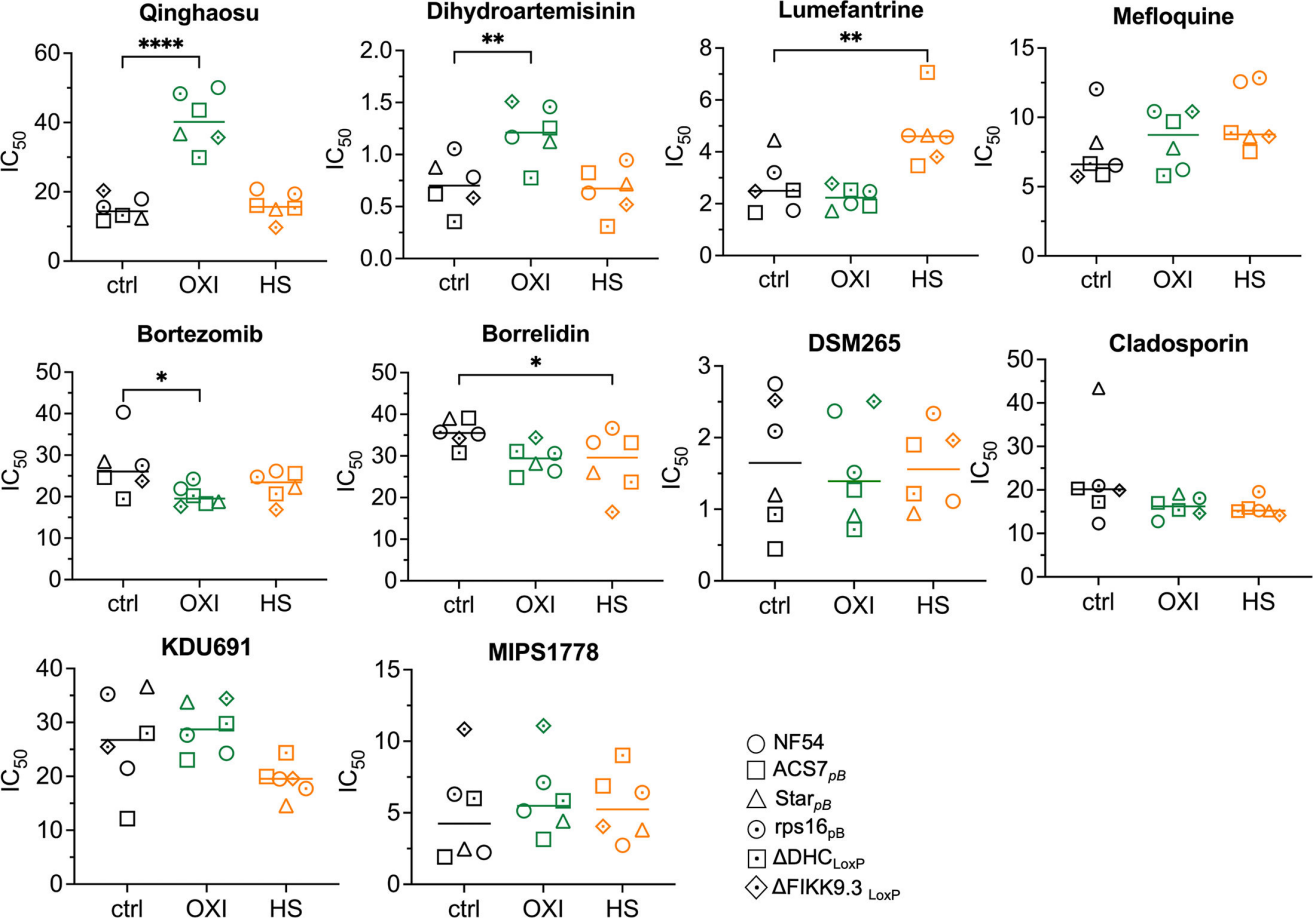
Chemosensitivity profiling of *P. falciparum* lines in oxidative stress microenvironment and heat-shock (HS) condition. Parasite line’s (different symbols) chemosensitivities (IC_50_ mean of biological replicates, Table S1) to each compound (Table S2) in each condition: control (ctrl), normal condition, oxidative stress (OXI), and HS. Differences between condition sets (“OXI vs ctrl” and “HS vs ctrl”) were examined using one-way analysis of variance followed by Dunett’s multiple comparison test. *P < 0.05, **P < 0.01, ****P < 0.0001.

IC_50_ growth responses of mutants to a wide range of compounds, oxi-treated, heat-shock, and untreated control (Table S2) allowed the assessment of possible chemosensitivity shifting of oxi-treated, heat-shock, and untreated RBCs (Fig. 2). The oxidative stress significantly decreased the artemisinin (QHS and DHA) efficacy (Fig. 1B), while heat shock significantly decreased the lumefantrine efficacy (Fig. 2). These findings suggest that the reduced effectiveness of artemisinin in oxidative stress is due to the activation of parasite stress responses, including upregulation of oxidative stress-related and antioxidant genes (4, 5, 7). Furthermore, the oxidative stress-induced tolerance to artemisinin was also significantly observed in parasites carrying mutations in genes involved in nuclear DNA repair (rhp_16_), regulating cytoskeleton (DHC), exported protein phosphorylation (FIKK9.3), and phospholipid transport (star-related) (Fig. 1B and 2).

Surprisingly, exposure to heat-shock parasites did not alter sensitivity to artemisinin, but there was a significant reduction in sensitivity to lumefantrine, which is an important partner drug in currently available artemisinin combination therapies (18) (Fig. 2). Previous studies revealed similarities between the processes vital for parasite survival of febrile temperatures and artemisinin (10). However, the mechanism by which the parasite responds to elevated oxidative stress, providing protection against artemisinin, appears not to be identical to induction of the parasite’s fever response. In contrast, increased expression of antioxidant-related genes and unfolded protein response genes associated with fever responses (10, 19) does enhance parasite tolerance to lumefantrine.

While oxidative stress from artemisinin treatment is associated with upregulation of unfolded protein response (9, 20) and general shutdown of protein synthesis (20, 21), our data confirm that BTZ provides a synergistic effect to artemisinin mechanism of action (22), increasing significantly the sensitivity of parasites’ oxidative stress exposure (Fig. 2). In addition, our data indicated a basal level of nucleic acid synthesis and translation remains active and represents potential synergistic target to counter artemisinin mechanisms of resistance. *De novo* pyrimidine synthesis and tRNA acylation (e.g., threonyl-tRNA and lysyl-tRNA synthetase [PfKRS]) (Table S1) appear to be attractive targets to counter parasite mechanisms that lower its sensitivity to the toxicity of artemisinin. For example, sensitivity to borrelidin, a putative inhibitor of threonyl-tRNA synthetase, increased in parasites exposed to heat shock (Fig. 2). Compounds such as DSM265 and cladosporin, which have been shown as effective inhibitors of these processes in *Plasmodium* species (Table S1), are not significant sensitive to pre-induced oxidative stress or heat shock-exposed parasite (Fig. 2). This finding may offer an additional potential combination therapy for malaria treatment due to their independent mechanism of action from artemisinin, once Lumefantrine, a common artemisinin partner (18) significantly reduced the sensitivity of parasites exposed to heat shock (Fig. 2). Of importance, DSM265, a potent inhibitor of the pyrimidine biosynthetic enzyme dihydroorotate dehydrogenase (23), demonstrated a long half-life of up to 5 days in humans (23), though its clinical development was halted due to non-clinical toxicology, resistance selection, and complexity of formulation; cladosporin, an inhibitor of PfKRS, a central enzyme in protein translation, exhibits potential antimalarial activity in the nanomolar range (24, 25). Further studies are needed to optimize the efficacy, safety, and practical use of inhibitors of these targets as antimalarial drugs.

## SUPPLEMENTAL MATERIAL

Supplemental Material mbio.03169-23-s0001.pdf Detailed information on the methods and reagents used in the study.

Legends mbio.03169-23-s0002.docx Descriptions of supplemental data.

Table S1 mbio.03169-23-s0003.pdf Compounds used in the study.

Table S2 mbio.03169-23-s0004.xlsx Raw data for drug inhibition assays.

ASM does not own the copyrights to Supplemental Material that may be linked to, or accessed through, an article. The authors have granted ASM a non-exclusive, world-wide license to publish the Supplemental Material files. Please contact the corresponding author directly for reuse.
