# Supplementary material for: Oxidative stress changes the effectiveness of artemisinin in Plasmodium falciparum
Source: mBio. 2024 Feb 7;15(3):e03169-23. doi: 10.1128/mbio.03169-23 (PMC10936410; doi:10.1128/mbio.03169-23)
Supplement: Supplemental Material — Detailed information on the methods and reagents used in the study. [file mbio.03169-23-s0001.pdf]

Supplementary Material

Parasite culture maintenance.

The wild type *P. falciparum* NF54, all *pB*-mutants and LoxP-mutants were cultured in 4% hematocrit (O+ erythrocytes from Interstate blood bank, Memphis, TN) and 1% Albumax II in RPMI 1640 medium (Invitrogen) supplemented with 50 μ g/ml hypoxanthine (Sigma) and 25 mM HEPES (Invitrogen). The culture flasks were grown in an incubator with continuous flow of mixed gas (90% Nitrogen, 5% CO2 and 5% O2 respectively). *piggyBac* mutants were generated on the NF54 clone background as described previously (Balu et al. 2009; Balu, Singh, Maher, and Adams 2010).

piggyBac mutant parasites

The description for creation and characterization of *piggyBac* mutants for PF3D7_1200700 ($ACS7_{pb}$), PF3D7_1250800 ($rhp16_{pb}$), and PF3D7_0104200 ($Star_{pb}$) were described previously (Balu et al. 2009; Balu, Singh, Maher, and Adams 2010; Zhang et al. 2021; Bronner et al. 2016).

Generation of LoxP parasites and conditional loxP Knockouts (KO)

To generate a LoxPint plasmids pL_FIKK9.3HA-T2A-NL_*GD, pL-FIKK*10.1-HA (Jones et al. 2016) vector kindly provided by Mike Blackman of Crick Institute, was used as backbone. The target gene FIKK9.3 homology fragment (1-963 nucleotides of gene with introns) was amplified from *P. falciparum* NF54 genomic DNA using primers JA4046 F: acgtaacagacttaggaggaATGAAATTTATTGGAGTGTTATTTTTA, JA4049 R: gttattgtatattatttttttatttacCATCTCTTGAACTCTACCA. The *P.* falciparum FIKK9.3 kinase domain (geneID) gene was recodonized, which corresponds to FIKK9.3 nucleotides 964 to 2165

lacking the 3’ native intron and stop codon. The recodonized kinase domain was synthesized (GenScript USA Inc.). BglII restriction site ligated with LoxPint sequence (AGATCTgtaaataaaaaaaataatatacaATAACTTCGTATAGCATACATTATACGAAGTTATtatata tgtatatatatatatatttatatattttatattcttttag) was flanked 5’ end of recodonized section, part of 3HA tag with BseEI site sequence (atgtacccatatgatgtaccggattacgcaggttatccgtatgacgtTCCGGA) was added 3’end of recodonized section. This BglII-FIKK9.3 recodonized section-HA-BspEI cassette was moved in a carrier vector pUC57, then sub cloned it into pfikk 10.1-HA vector. The homology fragments were then seamless ligated in plasmid pL_FIKK9.3rHA-T2A-NL_GD between BglII and BspEI restriction sites by GIBSON “DELETION” ASSEMBLY to create the plasmid pL_FIKK9.3HA-T2A-NL_GD. This plasmid contains: *P. falciparum* FIKK 9.3 (Gene ID: PF3D7_090220) homology region positioned 1-963 nucleotides of gene with introns, a LoxPint module flanked the 5’of the recodonized *P. falciparum* FIKK 9.3 kinase domain region positioned 964-2165 nucleotides of gene coordinating 287-633 aa of protein without introns, followings are fusion 3HA tags, Neomycin CDS, T2A self-cleaving peptides sequence, following then by the second loxPint modules flanked the 3’ end of T2A sequence, and the GFP gene and drug selectable marker hDHFR under the constitutive 5’CAM promoter and 3’HRP2 UTR.

To generate the LoxPint plasmid pL_DHC-HA-T2A-NLHA_NbEDG targeting *P. falciparum* DHC (PF3D7_1122900) gene (Figure S1-B), the LoxPint plasmids pL_FIKK9.3HA-T2A-NL_*GD* was used as backbone. First the homology region of FIKK9.3 was substituted by DHC homology region (673nt, coordinate 14272-14945), which was amplified from *P. falciparum* NF54 genomic DNA using primers (forward cgtaacagacttaggaggagatctCGGACAATATGATGATAAATTAAAAACAGTAATATTGGGTC and reverse ATtgtatattatttttttatttacCATATATTCAATTTCGGCATTTATGTGTAACCCT), and

then the recodonized region of FIKK9.3 was substituted by the recodonized region of DHC (synthetized by GenScript USA Inc.) (1366 nt, coordinate 14936-16302). Both regions were replaced used GIBSON "DELETION" ASSEMBLY. Specifically for this plasmid pL_DHC-HA-T2A-NLHA_NbEDG, addition GFP nano luciferase reporters' genes were included. The vector is designed to express both GFP and Nano luciferase driven by *P. berghei* EF1α (Figure S1-B), which is a constitutive promoter active in all *P. falciparum* developmental stages.

P. falciparum clone NF54-DiCre (Jones et al. 2016) and the transfected parasite lines established here were cultured as described (Jones et al. 2016). Routine synchronization was by Percoll enrichment and sorbitol treatment. For transfection, either purified schizont-stage or ring-stage parasites (> 5% parasitemia) were direct electroporation using an Amaxa 4D-Nucleofector. Selection with 5 nM WR99210 (Sigma-Aldrich) starts the day after transfection (day 1) for 5 days. Wait and let the parasitemia come back to ~1% (3 weeks), and drug again for 3 days with 5 nM WR99210. Once parasitemia comes back again (1 week), select for plasmid integration into the target gene locus with 250ug/ml G418 for 14 days, then stop until a viable parasite population was reestablished (~4 weeks). Anti-folate and G418 resistant clones were isolated by limiting dilution. Clones carrying integrated LoxPint plasmids were identified by PCR at both 5' and 3' -integration sites.

To induce DiCre-driven loxP site recombination, synchronized ring-stage parasites were treated with 100 nM RAP (Sigma) or DMSO only (final concentration 1% v/v) for 4 h. Parasites were subsequently washed twice with warm RPMI and returned to culture. Samples used for nucleic acid extraction were taken at least 24 h after rapamycin treatment, and samples used for PCR were taken at the end of the same asexual cycle (~44 h following RAP treatment) or in subsequent cycles.

Primers used in the constructions:

PRIMER ID	SEQUENCE
JA4082	ATTAAATCGGTTCAATTATGATTCTATAGAATAATGAA
JA4083	TGATGTGAATTATGAAAGTTGGATTTTGC
JJA4084	GTACTTCTTCATCAGGTTGTACTGCTTAA
JA4087	CCAGTAGTGCAAATAAATTTAAGGGTAAGTT
JA4097	AACGCAATTAATGTGAGTTAGCTCACTCATTA
JA4100	TGCAACCATAAATTTGTCAGCAGCTAAAAC
JA4230	TAAGAAAAACGAACATTAAGCTGCCATATCC
JA4234	ATACCAAATGAAAAAGATACCAAATTATTACCCGAA
JA4246	TCACCAGGTTTTTCTTTCTTTATTATTTTCGT

Red blood cells (RBC) oxidative stress pre-treatment

Methods for oxidative pre-treatment of hRBCs were as published previously (Cyrklaff et al. 2016),and used in Zhang et al (Zhang et al. 2021). Briefly, O + hRBCs (Interstate blood bank, packed, 100% hematocrit) were incubated with 1mM H_2O_2 (Sigma-Aldrich, Cat. no. H1009-100ML) for 1 h at room temperature. After treatment, cells were washed three times with phosphate buffered saline (PBS) before dithiothreitol (DTT) was added to a final concentration of 1mM to heal any reversible oxidative damages. Cells were then treated with menadione sodium bisulfite for one hour at room temperature (Sigma- Aldrich Cat. no. M5750-100G) and washed five times. A volume of 3–4ml of AB medium (RPMI 1640 medium supplemented with 2 mM L-glutamine, 25mM HEPES, 100 μM hypoxanthine, and 20 $\mu g ml^{-1}$ gentamicin) was added on top of the cell pellet after discarding the final wash. Pre-treated erythrocytes were stored at 4 °C before use in parasite culture.

Scalable drug assay standardization

Drug plates were pre-made in DMSO to run with the V&P Scientific 40 nL Pin Tool mounted on the Cybio Felix liquid handling robot. Each compound (Table S1) was serially diluted 1:2 along 12 columns of a 384 well plate constituting the master plate. Replicate daughter plates to be used with the Pin tool were made from the master plate. Daughter plates were stored at -20ºC and used only once.

The drug assay was a modification of the standard SyberGreen 72 hours drugs assay. The parasite lines were highly synchronized in schizonts stage using gradient percoll. Two groups of each parasite-line were split equally in 3 flasks: oxi group (contain pre-treated RBCs), heat-shock (HS) group (contain untreated RBCs) and the control group (contain untreated RBCs). Both

parasite-groups were incubated in continuous flow of mixed gas (90% Nitrogen, 5% CO2 and 5% O2 respectively) for ~16 hours, until the culture show >90% rings. The HS group was incubated for 4 hours at 41°C mimic the fiver condition. Then, the parasitemia for each parasite-line groups were adjusted for 0.4%. Parasites were spread in drug plate assays and grown for 72hours. The growth response for each compound dilution was obtained by reading the fluorescence generated by DNA intercalating dye SYBRGreen I (invtitrogen)(Johnson et al. 2007). Plates were analyzed by first reading on the CLARIOstar plate reader for relative fluorescence units (RFUs) at the optimal SybrGreen emissions (Ex/Em 484-15/528-15) and relative IC50 values were calculated by interpolation of the probit transformation of the log(dose)-response curve. Each batch of mutant assays were accompanied with wild type NF54. All compounds were performed in 3 to 4 replicates (Table S2).

To obtain the shift between oxi-groups' and control-groups' IC50 obtained for each parasite-line group was normalized by NF54-control of each correspondent compound. All statistic test was performed on Prism GraphPad (Version 9). The plots were obtained heatmp.2 package in R and Prism GraphPad.

Figures

Figure S1

A)

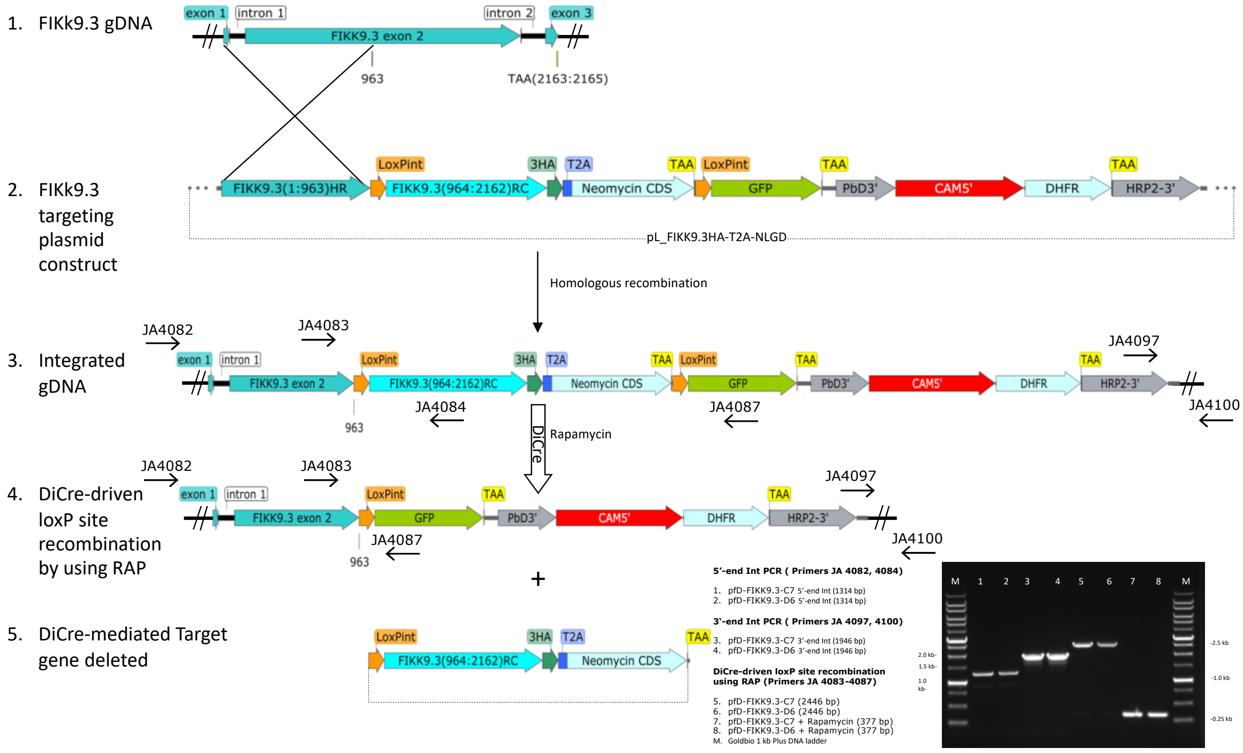

B)

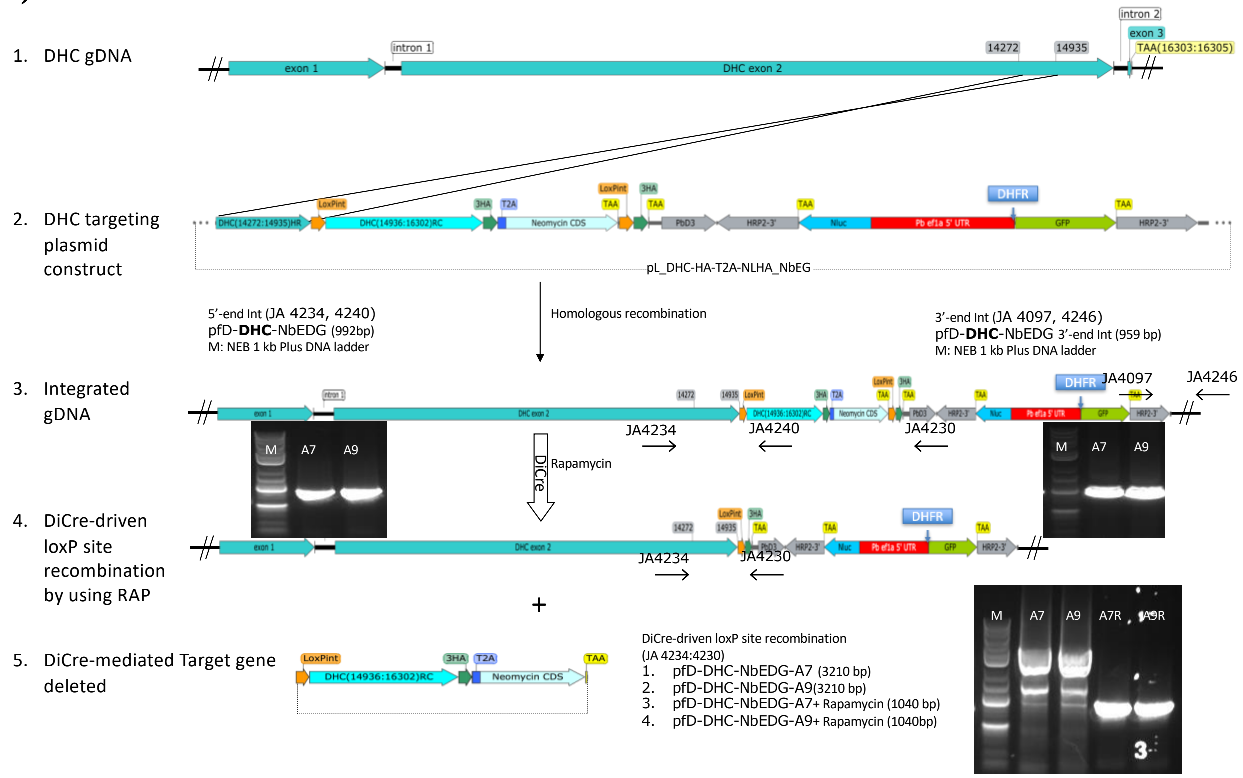
1. DHC gDNA
exon 1
intron 1
DHC exon 2
14272
14935
intron 2
exon 3
TAA(16303:16305)
2. DHC targeting plasmid construct
DHC(14272:14935)HR
LoxPint
DHC(14936:16302)RC
3HA
T2A
Neomycin CDS
TAA
PbD3
HRP2-3'
Nluc
Pb ef1a 5' UTR
DHFR
GFP
pL_DHC-HA-T2A-NLHA_NbEG
5'-end Int (JA 4234, 4240)
pfD-DHC-NbEDG (992bp)
M: NEB 1 kb Plus DNA ladder
Homologous recombination
3'-end Int (JA 4097, 4246)
pfD-DHC-NbEDG 3'-end Int (959 bp)
M: NEB 1 kb Plus DNA ladder
3. Integrated gDNA
JA4097
JA4246
JA4234
JA4240
JA4230
M
A7
A9
DiCre
Rapamycin
4. DiCre-driven loxP site recombination by using RAP
+
5. DiCre-mediated Target gene deleted
DiCre-driven loxP site recombination
(JA 4234:4230)
1. pfD-DHC-NbEDG-A7 (3210 bp)
2. pfD-DHC-NbEDG-A9(3210 bp)
3. pfD-DHC-NbEDG-A7+ Rapamycin (1040 bp)
4. pfD-DHC-NbEDG-A9+ Rapamycin (1040bp)
A7R
A9R

Figure S2

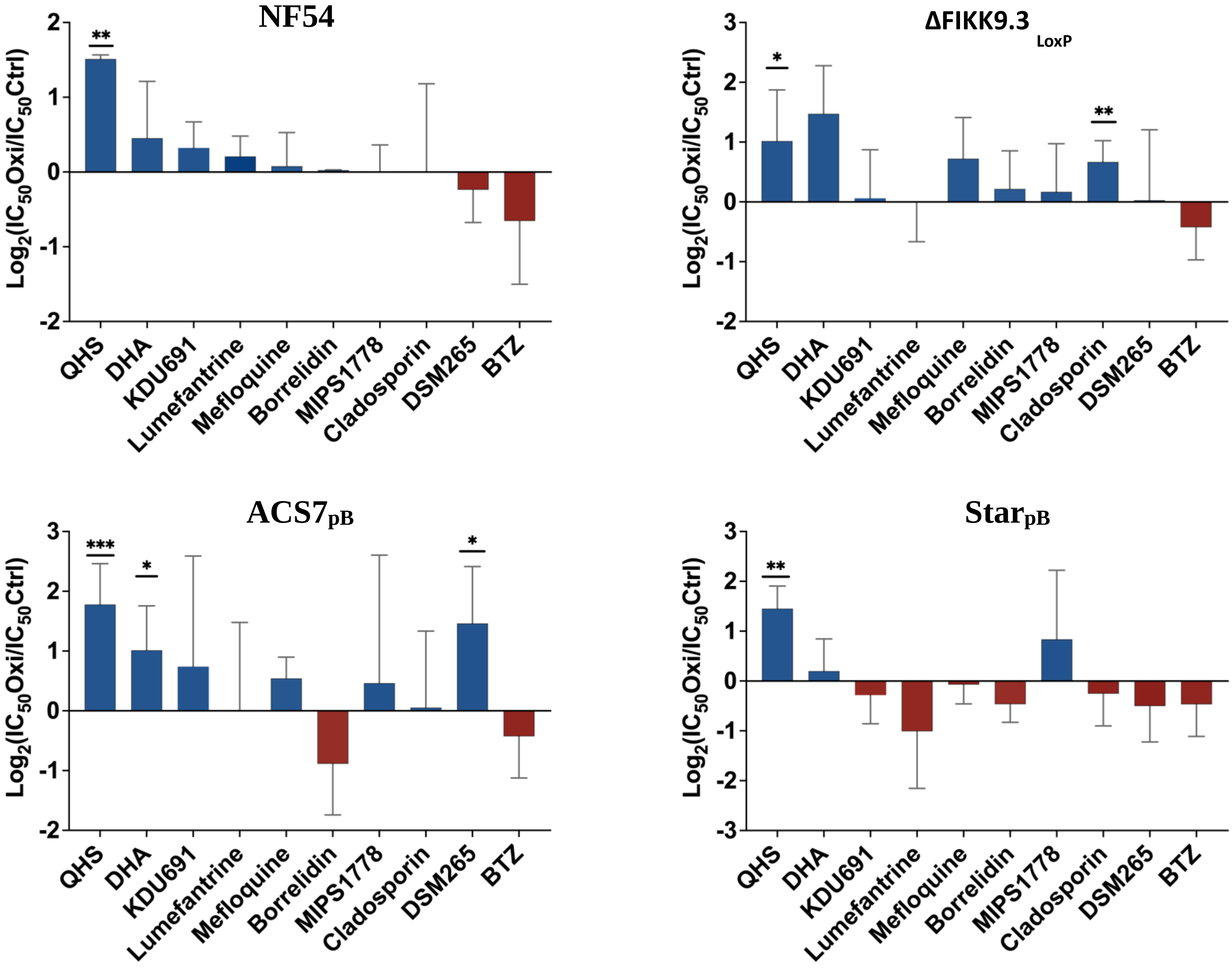
NF54
ΔFIKK9.3 LoxP
ACS7pB
StarpB
Log2(IC50Oxi/IC50Ctrl)
QHS
DHA
KDU691
Lumefantrine
Mefloquine
Borrelidin
MIPS1778
Cladosporin
DSM265
BTZ
*
**
***
3
2
1
0
-1
-2
-3

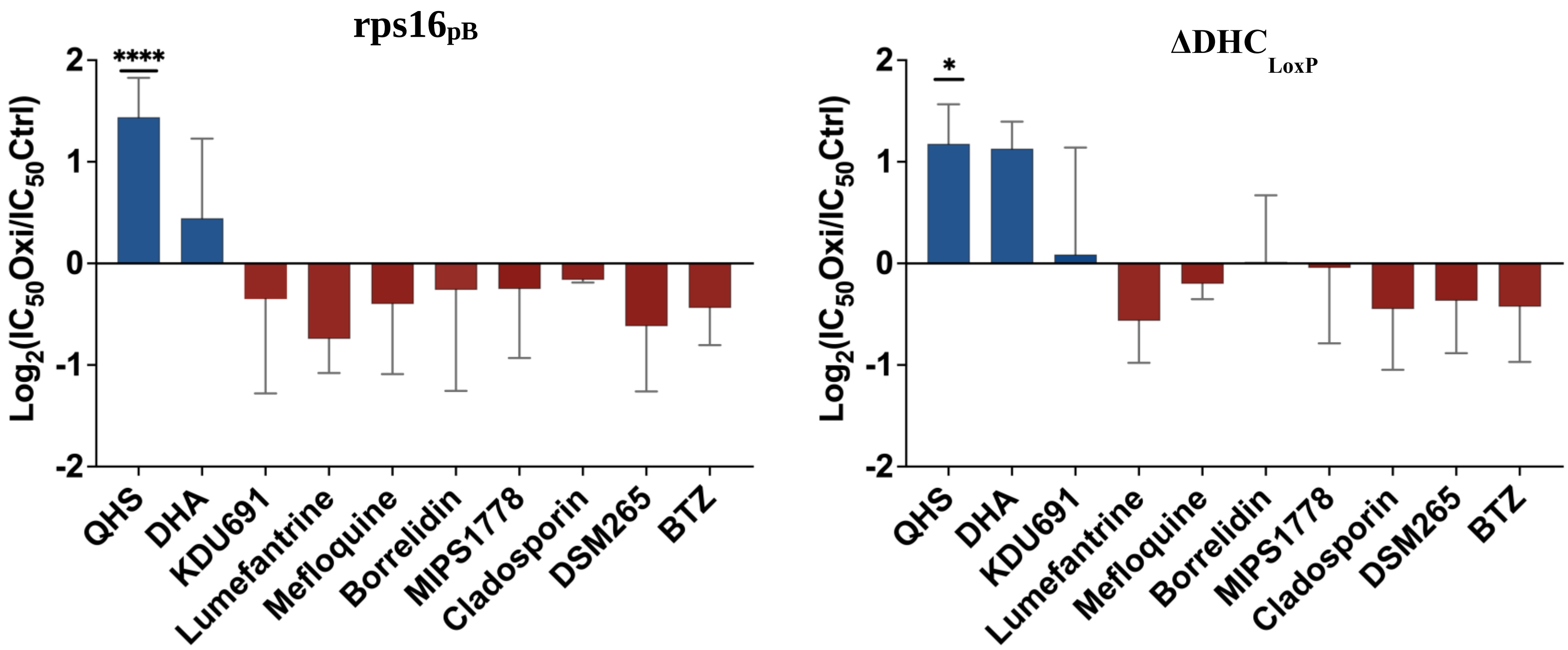

Chemosensitivity shifting of all line *P. falciparum*. The oxi-group was normalized by control-group of each corresponding compound, in each replicate. Error bar is corresponded to standard deviation. All statistical tests were performed on Prism GraphPad.
(* $p<0.05$, ** $p<0.01$, *** $p<0.001$, **** $p<0.0001$, Welch's t-test, IC50-Oxi-treated vs IC50-crtl-untreated of each drug)

References

Balu, B., C. Chauhan, S. P. Maher, D. A. Shoue, J. C. Kissinger, M. J. Fraser, and J. H. Adams. 2009. 'piggyBac is an effective tool for functional analysis of the Plasmodium falciparum genome', *Bmc Microbiology*, 9.

Balu, B., N. Singh, S. P. Maher, and J. H. Adams. 2010. 'A Genetic Screen for Attenuated Growth Identifies Genes Crucial for Intraerythrocytic Development of Plasmodium falciparum', *Plos One*, 5.

Bronner, I. F., T. D. Otto, M. Zhang, K. Udenze, C. Wang, M. A. Quail, R. H. Jiang, J. H. Adams, and J. C. Rayner. 2016. 'Quantitative insertion-site sequencing (QIseq) for high throughput phenotyping of transposon mutants', *Genome Res*, 26: 980-9.

Cyrklaff, M., S. Srismith, B. Nyboer, K. Burda, A. Hoffmann, F. Lasitschka, S. Adjalley, C. Bisseye, J. Simpore, A. K. Mueller, C. P. Sanchez, F. Frischknecht, and M. Lanzer. 2016. 'Oxidative insult can induce malaria-protective trait of sickle and fetal erythrocytes', *Nat Commun*, 7: 13401.

Johnson, J. D., R. A. Dennull, L. Gerena, M. Lopez-Sanchez, N. E. Roncal, and N. C. Waters. 2007. 'Assessment and continued validation of the malaria SYBR green I-based fluorescence assay for use in malaria drug screening', *Antimicrob Agents Chemother*, 51: 1926-33.

Jones, M. L., S. Das, H. Belda, C. R. Collins, M. J. Blackman, and M. Treeck. 2016. 'A versatile strategy for rapid conditional genome engineering using loxP sites in a small synthetic intron in Plasmodium falciparum', *Sci Rep*, 6: 21800.

Zhang, M., C. Wang, J. Oberstaller, P. Thomas, T. D. Otto, D. Casandra, S. Boyapalle, S. R. Adapa, S. Xu, K. Button-Simons, M. Mayho, J. C. Rayner, M. T. Ferdig, R. H. Y. Jiang, and J. H. Adams. 2021. 'The apicoplast link to fever-survival and artemisinin-resistance in the malaria parasite', *Nat Commun*, 12: 4563.
