# Supplementary material for: Oxidative stress changes the effectiveness of artemisinin in Plasmodium falciparum
Source: mBio. 2024 Feb 7;15(3):e03169-23. doi: 10.1128/mbio.03169-23 (PMC10936410; doi:10.1128/mbio.03169-23)
Supplement: Legends — Descriptions of supplemental data. [file mbio.03169-23-s0002.docx]

**Legend of supplementary data**

**Supplementary Material.**

- Additional methodology description, including additional references.
- **Figure S1**: LoxP parasites and conditional loxP Knockouts (KO) strategy of A) pL_FIKK9.3HA-T2A-NL_GD and B) pL_DHC-HA-T2A-NLHA_NbEDG
- **Figure S2**: Chemosensitivity shifting of all line P. falciparum. The oxi-group was normalized by control-group of each corresponding compound, in each replicate. Error bar is corresponded to standard deviation. All statistical tests were performed on Prism GraphPad. (* p<0.05, ** p<0.01, *** p<0.001, **** p<0.0001, Welch's t-test, IC50-Oxi-treated vs IC50-crtl-untreated of each drug)

**Table S1:** Compounds used in the study. Compound ID, name, Structure and the known Mechanism of Action (references below).

**Table S2:** Mean of half-maximal inhibitory concentrations (IC50), Standard deviation (SD) and number of biological replicates (n) obtained for each compound, each parasite line in each condition (oxidative stress – oxi, heat-shock – HS and control – ctrl). Parasite line: NF54 (wild-type), piggyBac mutants (ACS7 – pB, Rhp16 – pB, Star -pB),LoxP tag integrated mutants (DHC- int – LoxP, FiKK9.3-Int-LoxP, LoxP knock-out mutants (ΔDHC-LoxP, ΔFiKK9.3-LoxP).
