# Supplementary material for: Oxidative stress changes the effectiveness of artemisinin in Plasmodium falciparum
Source: mBio. 2024 Feb 7;15(3):e03169-23. doi: 10.1128/mbio.03169-23 (PMC10936410; doi:10.1128/mbio.03169-23)
Supplement: Table S1 — Compounds used in the study. [file mbio.03169-23-s0003.pdf]

Table S1: Compounds used in the study.

**Compound ID**	**Compound name, class or information**	**Structure**	**Mechanism of Action**
QHS	Artemisinin	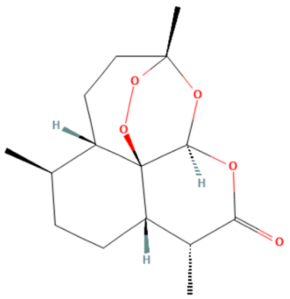	Inhibits parasite-mediated heme detoxification, generates intraparasite reactive oxygen species (ROS), and nonspecifically alkylates parasite proteins hemoglobin (Birnbaum et al. 2020; Bridgford et al. 2018; Ng, Fidock, and Bogyo 2017; Zhang et al. 2017)
DHA	diihydroarteminsin	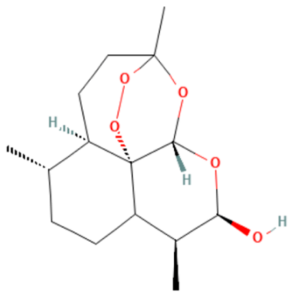	Inhibits parasite-mediated heme detoxification, generates intraparasite reactive oxygen species (ROS), and nonspecifically alkylates parasite proteins hemoglobin (Birnbaum et al. 2020; Bridgford et al. 2018; Ng, Fidock, and Bogyo 2017; Zhang et al. 2017)
BTZ	bortezomib	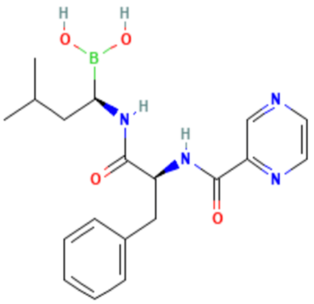	Proteasome inhibitor (Reynolds et al. 2007; Xie et al. 2021)
MMV000015	Mefloquine (racemic)	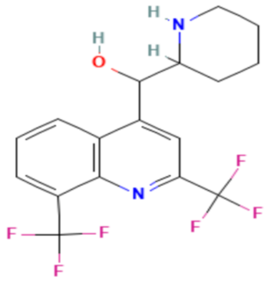	Targets 80S ribosome to inhibit protein synthesis (Wong et al. 2017)

MMV674143	Cladosporin - KRS	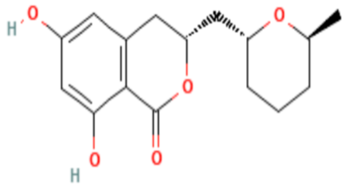	Inhibits lysyl-tRNA synthetase (PfKRS1)(Babbar et al. 2021; Baragana et al. 2019; Khan et al. 2014)
MMV155781 7	MIPS1778	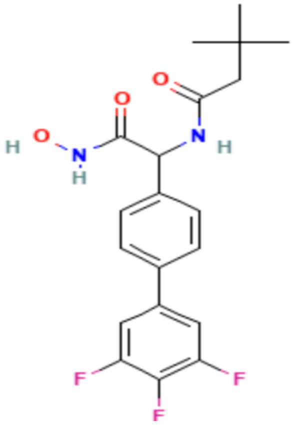	M17 aminopeptidase (+M1 potentially) inhibitor (Drinkwater et al. 2017)
MMV650381	KDU691 (Novartis)	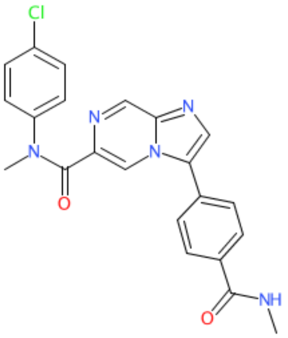	Phosphatidylinositol 4-kinase (PI(4)K) inhibitor (Dembele et al. 2017)
MMV000014	Lumefantrine	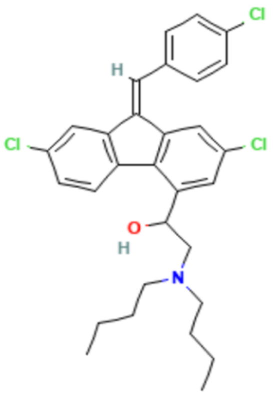	Inhibits the formation of β-hematin by forming a complex with hemin and inhibits nucleic acid and protein synthesis (de Villiers and Egan 2021)

MMV018912	DSM265	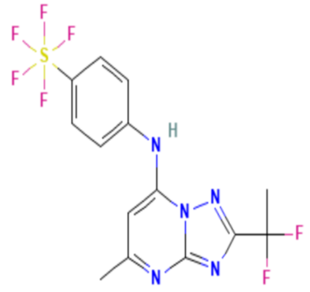	Dihydroorotate dehydrogenase (DHODH) inhibitor (Coteron et al. 2011; Phillips et al. 2015)
MMV084032	Borrelidin	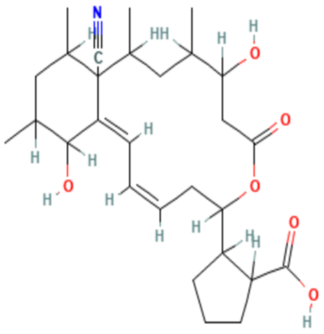	Threonyl-tRNA synthetase inhibits (Ishiyama et al. 2011; Novoa et al. 2014)

References

Babbar, P., P. Das, Y. Manickam, Y. Mankad, S. Yadav, S. Parvez, A. Sharma, and D. S. Reddy. 2021. 'Design, Synthesis, and Structural Analysis of Cladosporin-Based Inhibitors of Malaria Parasites', *ACS Infect Dis*, 7: 1777-94.

Baragana, B., B. Forte, R. Choi, S. Nakazawa Hewitt, J. A. Bueren-Calabuig, J. P. Pisco, C. Peet, D. M. Dranow, D. A. Robinson, C. Jansen, N. R. Norcross, S. Vinayak, M. Anderson, C. F. Brooks, C. A. Cooper, S. Damerow, M. Delves, K. Dowers, J. Duffy, T. E. Edwards, I. Hallyburton, B. G. Horst, M. A. Hulverson, L. Ferguson, M. B. Jimenez-Diaz, R. S. Jumani, D. D. Lorimer, M. S. Love, S. Maher, H. Matthews, C. W. McNamara, P. Miller, S. O'Neill, K. K. Ojo, M. Osuna-Cabello, E. Pinto, J. Post, J. Riley, M. Rottmann, L. M. Sanz, P. Scullion, A. Sharma, S. M. Shepherd, Y. Shishikura, F. R. C. Simeons, E. E. Stebbins, L. Stojanovski, U. Straschil, F. K. Tamaki, J. Tamjar, L. S. Torrie, A. Vantaux, B. Witkowski, S. Wittlin, M. Yogavel, F. Zuccotto, I. Angulo-Barturen, R. Sinden, J. Baum, F. J. Gamo, P. Maser, D. E. Kyle, E. A. Winzeler, P. J. Myler, P. G. Wyatt, D. Floyd, D. Matthews, A. Sharma, B. Striepen, C. D. Huston, D. W. Gray, A. H. Fairlamb, A. V. Pisliakov, C. Walpole, K. D. Read, W. C. Van Voorhis, and I. H. Gilbert. 2019. 'Lysyl-tRNA synthetase as a drug target in malaria and cryptosporidiosis', *Proc Natl Acad Sci U S A*, 116: 7015-20.

Birnbaum, J., S. Scharf, S. Schmidt, E. Jonscher, W. A. M. Hoeijmakers, S. Flemming, C. G. Toenhake, M. Schmitt, R. Sabitzki, B. Bergmann, U. Frohlke, P. Mesen-Ramirez, A. Blancke Soares, H. Herrmann, R. Bartfai, and T. Spielmann. 2020. 'A Kelch13-defined endocytosis pathway mediates artemisinin resistance in malaria parasites', *Science*, 367: 51-59.

Bridgford, J. L., S. C. Xie, S. A. Cobbold, C. F. A. Pasaje, S. Herrmann, T. Yang, D. L. Gillett, L. R. Dick, S. A. Ralph, C. Dogovski, N. J. Spillman, and L. Tilley. 2018. 'Artemisinin kills malaria parasites by damaging proteins and inhibiting the proteasome', *Nat Commun*, 9: 3801.

Coteron, J. M., M. Marco, J. Esquivias, X. Deng, K. L. White, J. White, M. Koltun, F. El Mazouni, S. Kokkonda, K. Katneni, R. Bhamidipati, D. M. Shackleford, I. Angulo-Barturen, S. B. Ferrer, M. B. Jimenez-Diaz, F. J. Gamo, E. J. Goldsmith, W. N. Charman, I. Bathurst, D. Floyd, D. Matthews, J. N. Burrows, P. K. Rathod, S. A. Charman, and M. A. Phillips. 2011. 'Structure-guided lead optimization of triazolopyrimidine-ring substituents identifies potent Plasmodium falciparum dihydroorotate dehydrogenase inhibitors with clinical candidate potential', *J Med Chem*, 54: 5540-61.

de Villiers, K. A., and T. J. Egan. 2021. 'Heme Detoxification in the Malaria Parasite: A Target for Antimalarial Drug Development', *Acc Chem Res*, 54: 2649-59.

Dembele, L., X. Ang, M. Chavchich, G. M. C. Bonamy, J. J. Selva, M. Y. Lim, C. Bodenreider, B. K. S. Yeung, F. Nosten, B. M. Russell, M. D. Edstein, J. Straimer, D. A. Fidock, T. T. Diagana, and P. Bifani. 2017. 'The Plasmodium PI(4)K inhibitor KDU691 selectively inhibits dihydroartemisinin-pretreated Plasmodium falciparum ring-stage parasites', *Sci Rep*, 7: 2325.

Drinkwater, N., J. Lee, W. Yang, T. R. Malcolm, and S. McGowan. 2017. 'M1 aminopeptidases as drug targets: broad applications or therapeutic niche?', *Febs Journal*, 284: 1473-88.

Ishiyama, A., M. Iwatsuki, M. Namatame, A. Nishihara-Tsukashima, T. Sunazuka, Y. Takahashi, S. Omura, and K. Otoguro. 2011. 'Borrelidin, a potent antimalarial: stage-specific inhibition profile of synchronized cultures of Plasmodium falciparum', *J Antibiot (Tokyo)*, 64: 381-4.

Khan, S., A. Sharma, H. Belrhali, M. Yogavel, and A. Sharma. 2014. 'Structural basis of malaria parasite lysyl-tRNA synthetase inhibition by cladosporin', *J Struct Funct Genomics*, 15: 63-71.

Ng, C. L., D. A. Fidock, and M. Bogyo. 2017. 'Protein Degradation Systems as Antimalarial Therapeutic Targets', *Trends Parasitol*, 33: 731-43.

Novoa, E. M., N. Camacho, A. Tor, B. Wilkinson, S. Moss, P. Marin-Garcia, I. G. Azcarate, J. M. Bautista, A. C. Mirando, C. S. Francklyn, S. Varon, M. Royo, A. Cortes, and L. Ribas de Pouplana. 2014. 'Analogs of natural aminoacyl-tRNA synthetase inhibitors clear malaria in vivo', *Proc Natl Acad Sci U S A*, 111: E5508-17.

Phillips, M. A., J. Lotharius, K. Marsh, J. White, A. Dayan, K. L. White, J. W. Njoroge, F. El Mazouni, Y. Lao, S. Kokkonda, D. R. Tomchick, X. Deng, T. Laird, S. N. Bhatia, S. March, C. L. Ng, D. A. Fidock, S. Wittlin, M. Lafuente-Monasterio, F. J. Benito, L. M. Alonso, M. S. Martinez, M. B. Jimenez-Diaz, S. F. Bazaga, I. Angulo-Barturen, J. N. Haselden, J. Louttit, Y. Cui, A. Sridhar, A. M. Zeeman, C. Kocken, R. Sauerwein, K. Dechering, V. M. Avery, S. Duffy, M. Delves, R. Sinden, A. Ruecker, K. S. Wickham, R. Rochford, J. Gahagen, L. Iyer, E. Riccio, J. Mirsalis, I. Bathhurst, T. Rueckle, X. Ding, B. Campo, D. Leroy, M. J. Rogers, P. K. Rathod, J. N. Burrows, and S. A. Charman. 2015. 'A long-duration dihydroorotate dehydrogenase inhibitor (DSM265) for prevention and treatment of malaria', *Sci Transl Med*, 7: 296ra111.

Reynolds, J. M., K. El Bissati, J. Brandenburg, A. Gunzl, and C. B. Mamoun. 2007. 'Antimalarial activity of the anticancer and proteasome inhibitor bortezomib and its analog ZL3B', *BMC Clin Pharmacol*, 7: 13.

Wong, W., X. C. Bai, B. E. Sleebs, T. Triglia, A. Brown, J. K. Thompson, K. E. Jackson, E. Hanssen, D. S. Marapana, I. S. Fernandez, S. A. Ralph, A. F. Cowman, S. H. W. Scheres, and J. Baum. 2017. 'Mefloquine targets the Plasmodium falciparum 80S ribosome to inhibit protein synthesis', *Nat Microbiol*, 2: 17031.

Xie, S. C., R. D. Metcalfe, H. Mizutani, T. Puhalovich, E. Hanssen, C. J. Morton, Y. Du, C. Dogovski, S. C. Huang, J. Ciavarri, P. Hales, R. J. Griffin, L. H. Cohen, B. C. Chuang, S. Wittlin, I. Deni, T. Yeo, K. E. Ward, D. C. Barry, B. Liu, D. L. Gillett, B. F. Crespo-Fernandez, S. Ottilie, N. Mittal, A. Churchyard, D. Ferguson, A. C. C. Aguiar, R. V. C. Guido, J. Baum, K. K. Hanson, E. A. Winzeler, F. J. Gamo, D. A. Fidock, D. Baud, M. W. Parker, S. Brand, L. R. Dick, M. D. W. Griffin, A. E. Gould, and L. Tilley. 2021. 'Design of proteasome inhibitors with oral efficacy in vivo against Plasmodium falciparum and selectivity over the human proteasome', *Proc Natl Acad Sci U S A*, 118.

Zhang, M., J. Gallego-Delgado, C. Fernandez-Arias, N. C. Waters, A. Rodriguez, M. Tsuji, R. C. Wek, V. Nussenzweig, and W. J. Sullivan, Jr. 2017. 'Inhibiting the Plasmodium eIF2alpha Kinase PK4 Prevents Artemisinin-Induced Latency', *Cell Host Microbe*, 22: 766-76 e4.
